# Research Infrastructure Core Facilities at Research Centers in Minority Institutions: Part I—Research Resources Management, Operation, and Best Practices

**DOI:** 10.3390/ijerph192416979

**Published:** 2022-12-17

**Authors:** Paul B. Tchounwou, Mohamad Malouhi, Elizabeth O. Ofili, Emma Fernández-Repollet, Daniel F. Sarpong, Richard Yanagihara, Renato J. Aguilera, Cecilia Ayón, Xiaoxin Chen, Asok Dasmahapatra, Song Gao, Cimona V. Hinton, Robert Holt, Vladimir Kolesnichenko, Michael D. Powell, Fatima Merchant, Kinfe K. Redda, Abiel Roche-Lima, Cecilia M. Shikuma, Jacqueline J. Stevens, Jose A. Torres, Robert T. Trotter, James Wachira, Paul Wang, Kristen J. Wells, Jason White, Yanyuan Wu

**Affiliations:** 1 RCMI Center for Health Disparities Research, Jackson State University, Jackson, MS 39217, USA; 2 Department of Clinical and Translational Sciences, Morehouse School of Medicine, Atlanta, GA 30310, USA; 3 Department of Pharmacology, School of Medicine, University of Puerto Rico Medical Sciences Campus, San Juan, PR 00936, USA; 4 Office of Health Equity Research, Yale University School of Medicine, New Haven, CT 06510, USA; 5 Department of Pediatrics & Department of Medicine, John A. Burns School of Medicine, University of Hawaii at Manoa, Honolulu, HI 96813, USA; 6 RCMI Border Biomedical Research Center, University of Texas at El Paso, El Paso, TX 79968, USA; 7 School of Public Policy, University of California-Riverside, Riverside, CA 92521, USA; 8 RCMI Center for Health Disparities Research, North Carolina Central University, Durham, NC 27707, USA; 9 Center for Biomedical and Minority Health Research, Texas Southern University, Houston, TX 77004, USA; 10 Department of Biological Sciences, Center for Cancer Research and Therapeutic Development, Clark Atlanta University, Atlanta, GA 30314, USA; 11 Department of Microbiology and Immunology, Meharry Medical College, Nashville, TN 37208, USA; 12 College of Pharmacy, Xavier University of Louisiana, New Orleans, LA 70125, USA; 13 Department of Engineering Technology, College of Technology, University of Houston, Houston, TX 77004, USA; 14 College of Pharmacy and Pharmaceutical Sciences, Florida Agricultural and Mechanical University, Tallahassee, FL 32307, USA; 15 Department of Basic Sciences, Ponce Health Sciences University, Ponce, PR 00716, USA; 16 Center for Health Equity Research, Northern Arizona University, Flagstaff, AZ 86011, USA; 17 RCMI Center for Urban Health Disparities Research and Innovation, Morgan State University, Baltimore, MD 21251, USA; 18 Department of Radiology, Howard University, Washington, DC 20059, USA; 19 Department of Psychology, San Diego State University, San Diego, CA 92182, USA; 20 RCMI Center for Biomedical Research, Tuskegee University, Tuskegee, AL 36088, USA; 21 Department of Internal Medicine, Charles R. Drew University, Los Angeles, CA 90095, USA

**Keywords:** RCMI program, research infrastructure core, laboratory management and operation, best practices

## Abstract

Funded by the National Institutes of Health (NIH), the Research Centers in Minority Institutions (RCMI) Program fosters the development and implementation of innovative research aimed at improving minority health and reducing or eliminating health disparities. Currently, there are 21 RCMI Specialized (U54) Centers that share the same framework, comprising four required core components, namely the Administrative, Research Infrastructure, Investigator Development, and Community Engagement Cores. The Research Infrastructure Core (RIC) is fundamentally important for biomedical and health disparities research as a critical function domain. This paper aims to assess the research resources and services provided and evaluate the best practices in research resources management and networking across the RCMI Consortium. We conducted a REDCap-based survey and collected responses from 57 RIC Directors and Co-Directors from 98 core leaders. Our findings indicated that the RIC facilities across the 21 RCMI Centers provide access to major research equipment and are managed by experienced faculty and staff who provide expert consultative and technical services. However, several impediments to RIC facilities operation and management have been identified, and these are currently being addressed through implementation of cost-effective strategies and best practices of laboratory management and operation.

## 1. Introduction

As mandated by the United States Congress, the Research Centers in Minority Institutions (RCMI) program was established in 1985 by the National Institutes of Health (NIH) to support academic institutions that offer doctoral degrees in the health professions or health-related sciences. In addition, institutions with a historical and current commitment to educating underrepresented students or providing health care services to medically underserved communities are also included [1]. Moreover, academic institutions eligible to apply for the RCMI program must have a 50 percent or greater enrollment of students from underserved communities (African Americans, Hispanics, Native Hawaiians, Pacific Islanders, Native Americans and Alaska Natives) that are underrepresented in the biomedical sciences [2,3].

The National Institute on Minority Health and Health Disparities (NIMHD) recognizes the significant contributions of minority-serving institutions to advancing scientific research, particularly on diseases or conditions that disproportionately impact racial/ethnic minorities and other U.S. populations that experience health disparities [4]. National statistics indicate that these institutions have played a key role in engaging minority populations in health sciences research and in the translation of scientific discoveries into culturally competent, measurable, and sustained improvements in health outcomes [5,6]. However, they often do not have adequate research resources to conduct and sustain cutting-edge biomedical, socio-behavioral, clinical, and/or translational research [4,5,6]. To address this issue in a cost-effective manner, the NIMHD provides cooperative agreement support to enable RCMI grantee institutions to address critical health issues, including cancer, cardiovascular disease, COVID-19, diabetes, HIV/AIDS, neurological disorders, and many other health problems plaguing underserved and underrepresented populations at disproportionately high rates [7]. The RCMI program also enables these institutions to become competitive in obtaining additional extramural support to foster further development and implementation of basic biomedical, behavioral, clinical, and/or translational research aimed at improving minority health and reducing or eliminating health disparities [1,8].

According to NIMHD, minority health is defined as all aspects of health and disease in one or more racial/ethnic minority populations. In contrast, health disparity is defined as a health difference based on one or more health outcomes (overall rate of disease incidence, prevalence, morbidity, mortality, survival, or quality of life) that adversely affect disadvantaged populations [4,9]. Current NIMHD-designated health disparity populations include Office of Management and Budget-defined racial/ethnic minorities, socioeconomically disadvantaged populations, underserved rural populations, and sexual and gender minorities (which include lesbian, gay, bisexual, transgender, and gender-nonbinary or gender-nonconforming individuals) [10]. Hence, health disparities research is a multi-disciplinary field of study devoted to gaining greater scientific knowledge about the influence of health determinants and defining mechanisms that lead to disparities, and how this knowledge is translated into interventions to reduce or eliminate adverse health differences [11].

**Figure 1:**
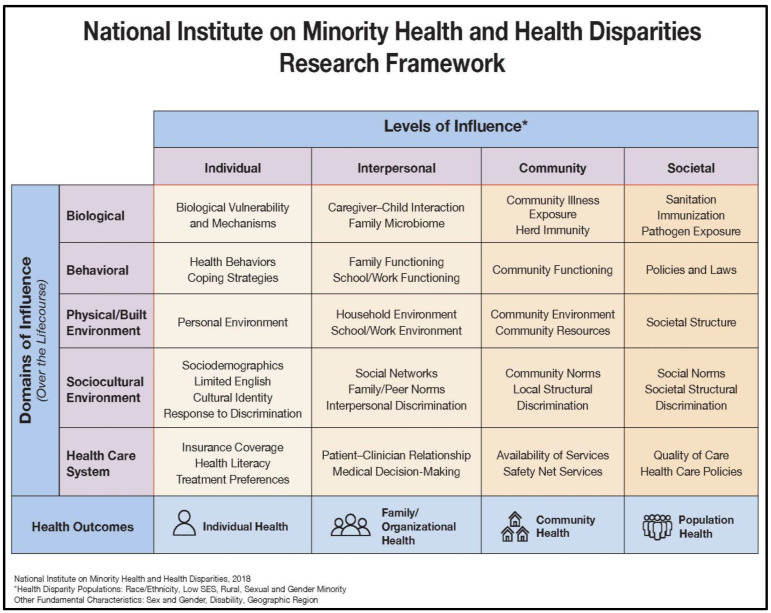
Established framework for minority health and health disparities research [4,13].

To support its research agenda, NIMHD has recently articulated nine strategic goals in its 2021–2025 Strategic Plan [12]. This plan serves as a blueprint to guide the science of minority and health disparities by implementing cost-effective strategies to accomplish the following scientific research goals: (1) Promote research to understand and to improve the health of racial/ethnic minority populations; (2) Advance scientific understanding of the causes of health disparities; (3) Develop and test interventions to reduce health disparities; (4) Create and improve scientific methods, metrics, measures, and tools that support health disparities research; (5) Support training to enhance diversity and to promote training and career advancement of minority health and health disparities researchers; (6) Strengthen the national capacity to conduct minority health and health disparities research; (7) Ensure appropriate representation of minority and other health disparity populations in NIH-funded research; (8) Promote evidence-based community engagement, dissemination, and implementation of minority health and health disparities research best practices; and (9) Cultivate and expand a community of minority health and health disparities researchers and advocates [12]. Figure 1 presents the established framework for minority health and health disparities research conceived by NIMHD [7]. The framework presents a comprehensive list of important key factors that are relevant to the science of minority health and health disparities. It highlights the complex and multi-faceted nature of minority health and health disparities, and, hence, serves as a strong catalyst for developing and implementing innovative research that spans different domains of influence (Biological, Behavioral, Physical/Built Environment, Sociocultural Environment, Healthcare System) as well as different levels of influence (Individual, Interpersonal, Community, Societal) within those domains [4,13].

Unlike the NIMHD-funded Centers of Excellence that focus on a thematic area of health disparities research, such as obesity, diabetes, etc. [14], RCMI U54 Centers have the flexibility to simultaneously address multiple health issues of concern to minority and underrepresented populations. The most recent funding opportunity announcement (RFA-MD-22-002) issued by NIMHD [15] requires each RCMI Specialized Research Center to have four major components:

(1) Administrative Core (AC) that provides strategic direction and administrative oversight to ensure that the strategic activities of the RCMI Program are implemented in a timely and cost-effective manner. AC also implements integrated governance and management structure, provides day-to-day financial management, facilitates the implementation of research conducted by leaders of both research and pilot projects, fosters collaborations and partnerships, facilitates the implementation of activities and services provided by other core components, and oversees the evaluation of all programmatic activities [15].
(2) Investigator Development Core (IDC) that provides financial support to promising early-stage investigators (ESIs) to implement innovative research projects that allow them to collect preliminary data needed to formulate new research hypotheses toward the development and submission of independent research proposals to address important health disparities issues of concern to minorities and underserved communities. IDC also has a built-in developmental component that allows ESIs (as well as post-doctoral research fellows, and early-career faculty) to sharpen their grantsmanship and other relevant research and scientific skills necessary to achieve biomedical research independence [15].
(3) Community Engagement Core (CEC) that serves as the nexus for the translation of novel research findings to reduce the incidence and prevalence of health disparity-related diseases by establishing long-term relationships with community-based organizations to address health disparity issues of concern to minority and underserved communities; facilitating their recruitment, engagement and retention as study participants in biomedical research, and disseminating the findings from both research and pilot projects into community-level practice and prevention to improve community health and reduce health disparities [15].
(4) Research Infrastructure (RIC), now called Research Capacity Core, provides an essential platform to enhance the quality and productivity of research projects and funded pilot projects, as well as to foster intra- and inter-institutional collaborations and partnerships between RCMI investigators and other biomedical, socio-behavioral, clinical, and/or translational researchers.

A U54 Center may also establish an optional Recruitment Core to hire seasoned investigators who have established track records of successful independent research support, as evidenced by NIH R-series, P-series, and/or U-series awards within the last two years, or other federal or non-federal awards, and who can serve as mentors to junior investigators [15]. Table 1 presents the specific characteristics of selected R-series, P-series, and U-series funding mechanisms [16].

NIMHD currently funds 21 RCMI institutions through U54 Specialized Center awards, with the primary goals to: “(1) enhance institutional research capacity to conduct world-class basic biomedical, behavioral, population and/or clinical research; (2) enable all levels of investigators at the recipient institution to become more successful in obtaining competitive extramural support, especially from NIH, for research on diseases and conditions that disproportionately impact populations that experience health disparities; (3) foster institutional environments conducive to research career development and enhancement for post-doctoral fellows, junior faculty, and other early-stage investigators; (4) enhance the tools for and conduct of research generally and specifically for improving minority health and reducing health disparities; and (5) establish sustainable partnerships with community-based organizations to promote research efforts and the dissemination of research findings” [15].

Moreover, to reduce some of the key impediments to collaborative research and to facilitate collaboration and team science, NIMHD has recently provided additional funding to support the establishment of a national RCMI Coordinating Center (RCMI-CC) that works closely with key personnel at all RCMI Specialized Centers and with NIMHD staff to help the centers collectively achieve their primary goals [17]. As such, the RCMI-CC serves as a national resource to advance the science of minority health and health disparities across the RCMI U54 Centers by coordinating intellectual exchange and collaborative interactions that enhance research capacity and competitiveness. In alignment with the goals of RCMI U54 Centers in four key domains, the RCMI-CC specific aims are to: (1) Coordinate program administration and evaluation across the RCMI U54 Centers; (2) Coordinate and leverage research resources to support scientific projects; (3) Coordinate mentoring and career development of new and early-stage investigators; and (4) Coordinate sustainable engagement with community organizations and partners.

Being one of the major core domains of the RCMI Program, the RIC is fundamentally important for biomedical and health disparities research. The RIC facilities are centralized shared research resources that provide access to sophisticated technologies and specialized instrumentation operated by technical personnel who provide scientific expertise and consultation services to health disparities researchers. As such, RIC resources are expected to provide faculty-level expertise in research methodology, specialized laboratory techniques, statistics, data science, biomedical/health informatics, community-engaged methods, and technical support in study design, biostatistics, and research ethics. They are also expected to foster collaborations and partnerships with other NIH supported centers, including those of the Clinical and Translational Science Awards (CTSA), Institutional Development Award (IDeA) Networks for Clinical and Translational Research (IDeA-CTR), Resource Centers for Minority Aging Research Centers (RCMAR), the Multiple Chronic Disease Research Centers (NIMHD), and other consortia [15].

The overarching goal of the RIC domain of the RCMI-CC is to coordinate and leverage research resources to support the development and cost-effective implementation of scientific projects including the research and pilot projects conducted by RCMI investigators. Hence, the RIC coordination creates a robust, inclusive ecosystem that provides access to novel and innovative resources widely throughout the existing RCMI U54 Centers while facilitating transdisciplinary capacity building across the RCMI Consortium. In addition to fostering research resources sharing and innovation, the RIC domain also offers opportunities for multi-disciplinary and transdisciplinary collaboration in biomedical, socio-behavioral, clinical, and/or translational research aimed at addressing minority health and reducing or eliminating health disparities. Therefore, the current study was designed to: (1) assess the biomedical, socio-behavioral, clinical, and translational research resources available at RCMI U54 Centers; and (2) evaluate the best practices in research resources management, operation and networking across the RCMI Consortium.

## 2. Materials and Methods

This research is a cross-sectional study based on the development of a comprehensive survey questionnaire that was administered to RIC facilities Directors and Co-Directors at 21 RCMI U54 Centers using the Research Electronic Data Capture (REDCap) system. Initially developed by a multi-institutional consortium of researchers at Vanderbilt University, Nashville, Tennessee, USA, REDCap is a secured web-based software that is widely used nationally and internationally for collecting, managing, and curating data from research studies. It is a very useful scientific tool that allows researchers to design web-based surveys and engage potential research participants through electronic communications. This technology also offers great flexibility for easy data manipulation with audit trails, reports for monitoring and querying participant records, and data analysis facilitated by an automated export to common statistical packages such as Statistical Package for Social Sciences (SPSS) and Statistical Analysis System (SAS) [18].

The server supporting the REDCap system was located in a secure facility with multiple layers of physical safeguards. Access to the server and research data was protected by a hardware-based firewall and a Virtual Private Network. Moreover, only authorized users had access to the REDCap system, which was password-protected. No personally identifiable information was included in the survey questionnaire. When all responses were received, summary reports of findings were generated and discussed shared with the RCMI Consortium.

Composed of a set of relevant questions, the questionnaire was emailed to all RIC Directors and Co-Directors who responded to the questions after consenting to participate in the study. In addition to multiple-choice and yes/no questions, open-ended questions were also developed to provide opportunity to the respondents to express their ideas and to reduce potential bias in selecting suggested answers.

The research protocol (No. 0030-22) for this project, entitled “RCMI Coordinating Center Research Infrastructure Cores Survey”, was reviewed and approved by the Institutional Review Board (IRB) at Jackson State University (JSU) on 28 February 2022. In addition, a multi-institutional agreement was signed by the official/authorized representatives of other RCMI U54 Centers allowing the JSU-IRB to review and provide appropriate oversight for the research project.

All participants provided an informed consent statement. The research survey was administered on 22 March 2022 and closed on 8 May 2022. A total of 57 Core Directors and Co-Directors responded to the REDCap survey. From the list of 98 Core Directors and Co-Directors of specific multi-user core laboratories at the 21 RCM U54 Centers, we estimated that a sample size of 49 would be adequate at the 95% confidence level and 10% confidence interval; using the freely available online sample size calculator provided by Creative Research Systems [18]. Hence, with 57 participants, the response rate of 58.2% was very acceptable. The SAS software was used to analyze the survey data. The research findings were explained using descriptive statistics based on several assessment metrics and indicators and presented graphically.

## 3. Results

### 3.1. Biomedical Research Resources at RCMI U54 Centers

**Figure 2:**
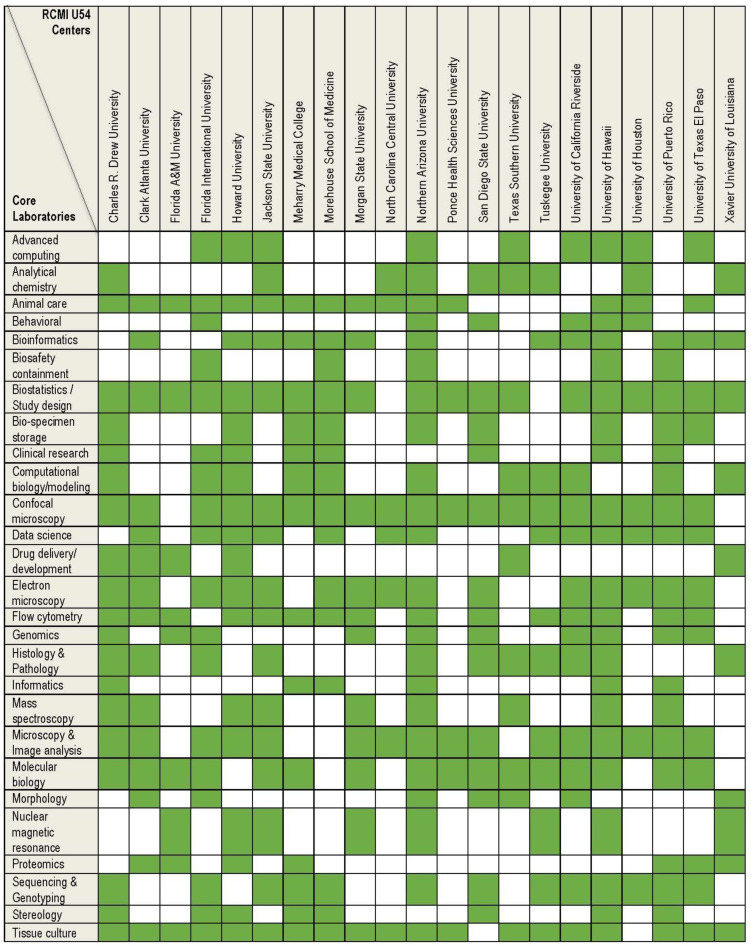
Research Infrastructure Core Facilities at the 21 RCMI U54 Centers.

Figure 2 highlights the specific core research facilities available at each of the 21 RCMI U54 Centers. It is evident from this figure that RCMI grantee institutions have established a significant number of multi-user facilities that enable their biomedical, socio-behavioral and/or clinical investigators to conduct innovative health disparities research.

The green boxes highlight the availability of core research facilities at these institutions of higher learning. The non-uniformed distribution of cores among the RCMI U54 Centers underscores the opportunities for research collaboration, and sharing of research resources across the RCMI Consortium.

### 3.2. Biomedical, Socio-Behavioral, and Clinical Research Equipment at RCMI U54 Centers

As shown in Table 2, the core facilities at RCMI U54 Centers are very well equipped with state-of-the-art instruments and other resources that allow them to perform specific key functions based on the type of research equipment and scientific software available and the technical services provided. Hence, the resources and services provided enhance the research infrastructure and enable the principal investigators of both research and pilot projects to develop and implement innovative biomedical, socio-behavioral and/or clinical research aimed at improving minority health and reducing/eliminating health disparities. Therefore, these core facilities play a critical role in designing robust experimental procedures and implementing comprehensive research approaches and methods to advance the science of minority health and health disparities.

### 3.3. Tenure Status of RIC Directors and Co-Directors at RMCI U54 Centers

**Figure 3:**
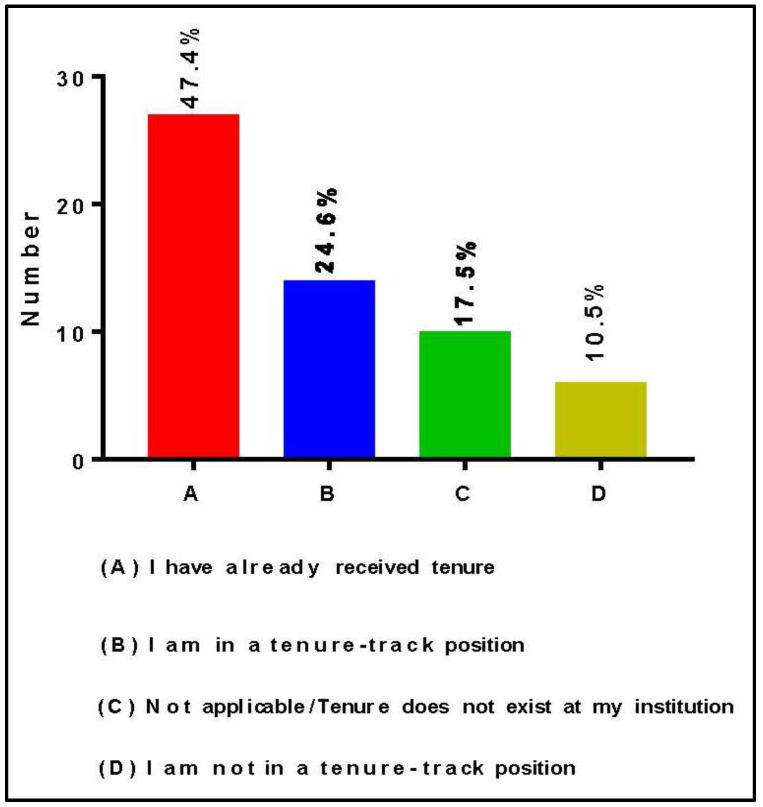
Tenure status of Directors and Co-Directors of RCMI RIC facilities.

As previously stated, the RIC is one of the four required core components of the RCMI Program at each RCMI U54 Center. Moreover, the RIC must be headed by a faculty leader who provides oversight [16]. Figure 3 presents the tenure status of Directors and Co-Directors of RCMI core research laboratories. Among the 57 respondents, 27 (47.4%) were tenured faculty, 14 (24.6%) were on tenure track, 10 (17.5%) work at institutions where tenure does not exist, and 6 (10.5%) were on non-tenure track positions.

### 3.4. Number of RIC Directors and/or Co-Directors at Each Specific RIC Laboratory at RMCI U54 Centers

Table 3 shows the numbers and percentages of Directors and Co-Directors of specific core research laboratories at RCMI U54 Centers. Among the 57 respondents to the survey questionnaire, 13 (22.8%) were the Directors of overall RIC facilities. Other respondents included seven (12.3%) Directors each for the Imaging Electron/Confocal/Fluorescence Microscopy Core, Bioinformatics Core, and Flow Cytometry Core; four (7.1%) for the Histology and Pathology Core, three (5.3%) each for the Biostatistics Core, Molecular Biology Core, Computational Biology/Chemistry Structure and Modeling Core Resources, and Investigator Development Core, Mass Spectroscopy Core; two (3.5%) each for the Genomics, Microarray, and RNASeq Core, and Mass Spectroscopy Core; two (3.5%) each for the Proteomics/Metabolomics Core, Animal Core, Analytical Chemistry Core, and Nuclear Resonance Imaging (NRI) and Nuclear Magnetic Resonance (MR) Spectroscopy Core; and one (1.8%) each for the Bio-Safety Containment Unit Core, and Drug Delivery/Development Core. In addition, 10 (17.5%) respondents indicated that they lead other types of cores, such as a Career Enrichment Program, Behavioral and Population Intervention Core,. Endocrine Core, CT, SPECT, PET and Optical Imaging Core, Wearable Sensor Instrumentation Core, Sensors Technologies Core, and Biomedical Informatics Core. As shown in Table 3, a total of 73 responses were collected. However, the percentage values presented on this table were calculated based on the fact that 57 RIC leaders and co-leaders participated in the survey. Hence, it is evident that some of the RIC leaders also serve as co-leaders of other specialized core laboratories.

**Table 3:** Numbers and Percentages of Survey Respondents who serve as Directors or Co-Directors of Specific Core Laboratories at the 21 RCMI U54 Centers.

Core Laboratory	Number of Respondents	Percentage of Respondents
Overall RIC facility	13	22.8%
Other, specify	10	17.5%
Imaging Core, Electron/Confocal/Fluorescence Microscopy	7	12.3%
Bioinformatics Core	7	12.3%
Flow Cytometry Core	7	12.3%
Histology and Pathology Core	4	7.1%
Biostatistics Core	3	5.3%
Molecular Biology Core	3	5.3%
Computational Biology/Chemistry Structure and Modeling Core Resources	3	5.3%
Genomics Core, Microarray, RNA seq, etc.	3	5.3%
Mass Spectroscopy Core	3	5.3%
Proteomics/Metabolomics Core	2	3.5%
Animal Care Core	2	3.5%
Analytical Chemistry Core	2	3.5%
Magnetic Resonance Imaging (MRI)/ Nuclear Magnetic Resonance (NMR) Core	2	3.5%
Bio-Safety Containment Unit Core, e.g., BSL-3	1	1.8%
Drug Delivery/Development Core	1	1.8%

### 3.5. Technical Services Provided by the Directors and/or Co-Directors of RIC Facilities

**Figure 4:**
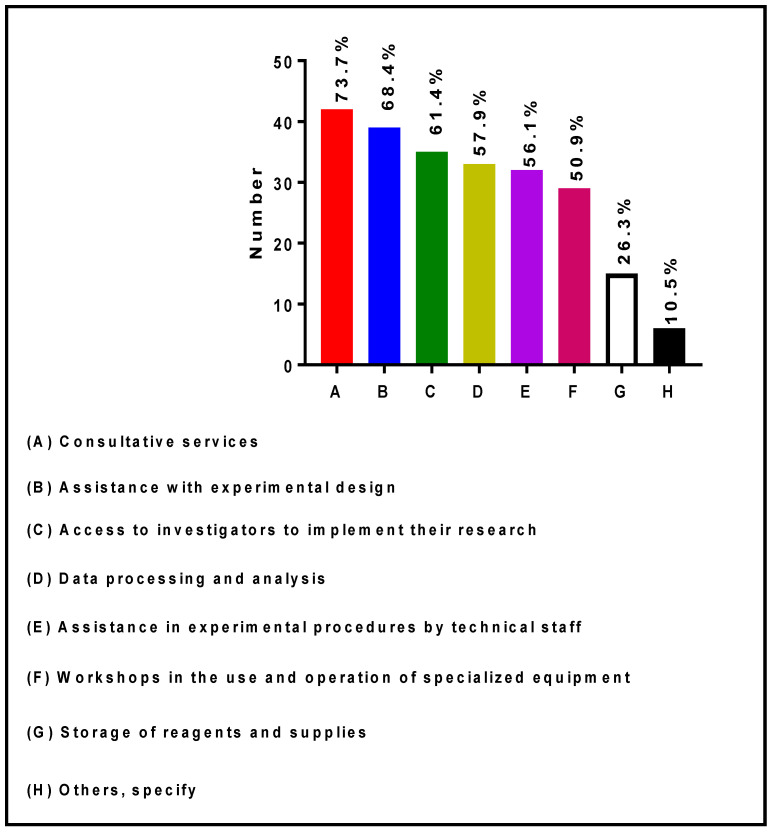
Technical services provided by RIC Directors and Co-Directors at RCMI U54 Centers.

Figure 4 presents the technical services provided by the Directors and/or Co-Directors of RIC facilities. The survey responses indicated that 42 (73.7%), 39 (68.4%), 35 (61.4%), 33 (57.9%), 32 (56.1%), 29 (50.9%), and 15 (26.3%) of Core Directors provide: Consultative services, assistance with experimental design, access to investigators to implement their research, data processing and analysis, assistance in experimental procedures by technical staff, workshops in the use and operation of specialized equipment, and storage of reagents and supplies, respectively. In addition, six (10.5%) Core Directors indicated that they provide other types of services such as: bench space, full access to equipment after training, research support workshops, research design and implementation, community collaboration, intervention methods, measurement methods, health sensor methods, IT services, wet laboratory, anthropometrics, and physical fitness testing resources, hands-on grant writing workshops, pilot grant funding, and/or summer collaborative sabbatical funding.

### 3.6. Outreach Activities Performed by RIC Directors and Co-Directors

**Figure 5:**
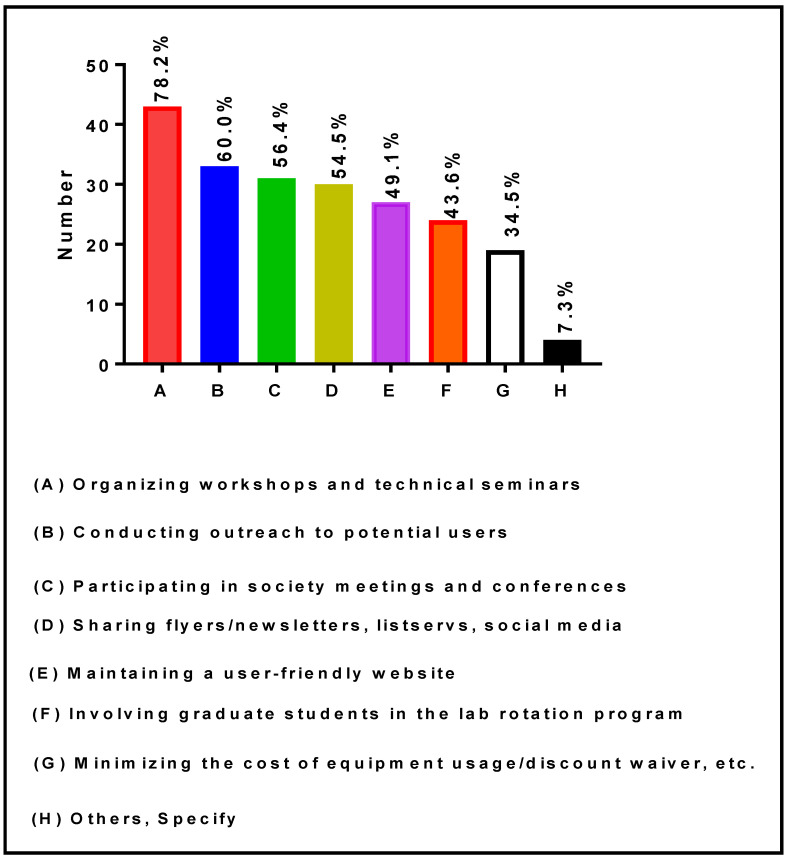
Outreach activities conducted by RIC Directors and Co-Directors at RCMI U54 Centers.

Figure 5 presents a comprehensive list of outreach activities carried out by RIC Directors and Co-Directors to bring new users to their core research facilities. It appears from this figure that several important outreach strategies are being implemented. Among the RIC Directors and Co-Directors, 43 (78.2%), 33 (60.0%), 31 (56.4%), 30 (54.5%), 27 (49.1%), 24 (43.6%), and 19 (34.5%) indicated that they: (a) Oganize workshops and technical seminars; (b) Conduct targeted outreach to potential users; (c) Participate in society meetings and conferences; (d) Share flyers/newsletters, listservs, social media; (e) Maintain a user-friendly website; (f) Involve graduate students in the lab rotation program; and (g) Minimize the cost of equipment usage and provide discount waivers, etc. Moreover, four (7.3%) indicated that they conduct grant-writing workshops and provide editorial service, conduct university-wide announcements and maintain a Canvas site for the Core, and/or are developing our recharge infrastructure to offer services outside the RCMI-funded projects.

### 3.7. Strategic Activities Implemented by RIC Directors and Co-Directors to Facilitate Access and Utilization of Scientific Equipment

**Figure 6:**
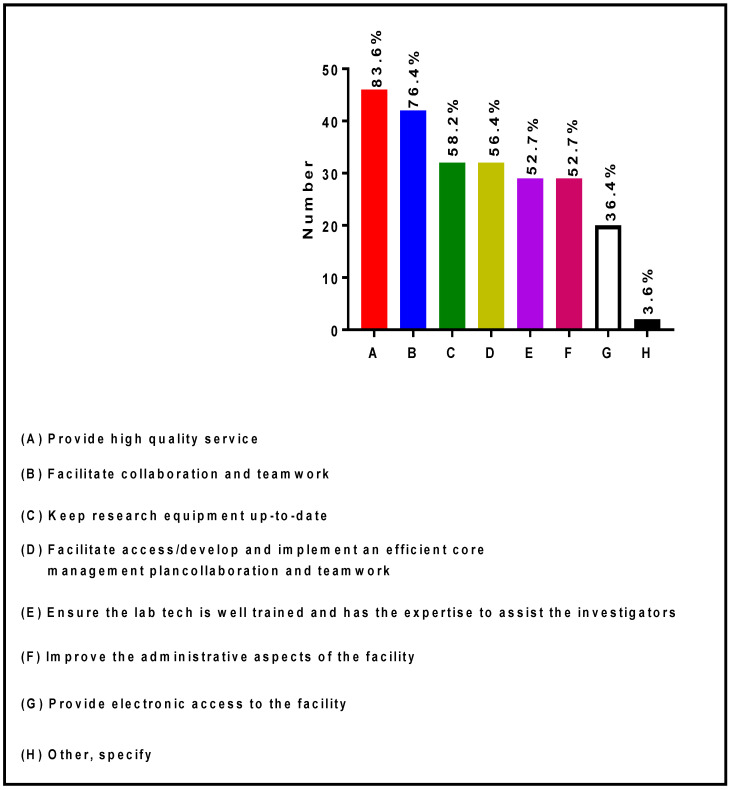
Strategic activities implemented by RIC Directors and Co-Directors to facilitate access to core laboratories.

Figure 6 describes the strategic activities implemented by RIC Directors and Co-Directors to facilitate access and utilization of scientific equipment. More than 50% of Directors and Co-Directors facilitate access to their core laboratories by: (1) Providing high-quality services; (2) Facilitating collaboration and teamwork; (3) Keeping research equipment up-to-date; (4) Developing and implementing an efficient core management plan; (5) Ensuring that the laboratory technicians are well trained and have the expertise to assist the investigators; and (6) Improving the administrative aspect of the core facilities. In addition, some Core Directors and Co-Directors provide electronic access to their core facilities.

### 3.8. Strategic Activities Implemented by RIC Directors and Co-Directors to Achieve Sustainability of Core Research Facilities

**Figure 7:**
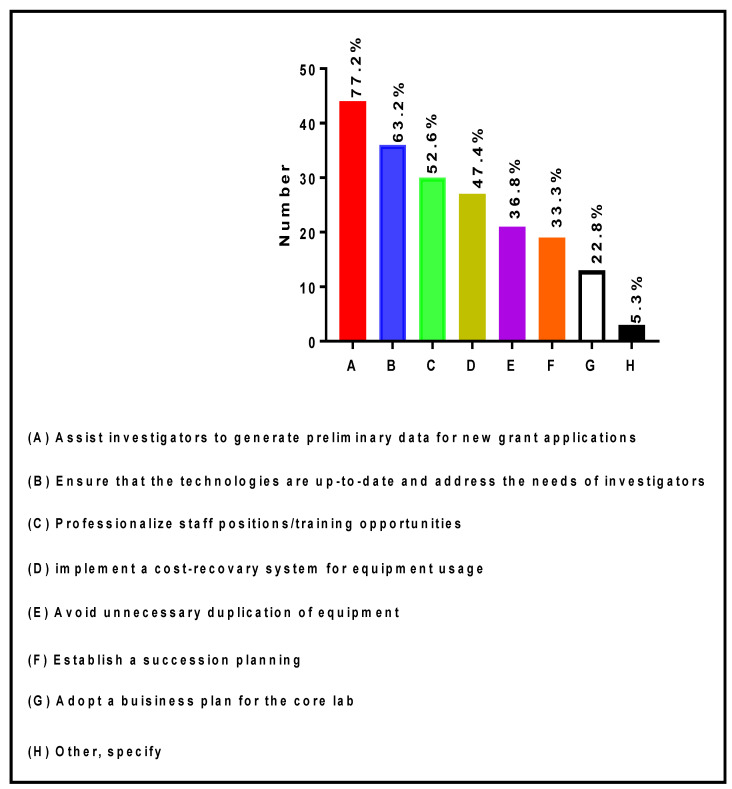
Strategic activities implemented by RIC Directors and Co-Directors to achieve sustainability of core laboratories.

RCMI RIC Directors and Co-Directors are utilizing several strategic approaches to achieve sustainability of their specific core laboratories. Among the survey respondents, 44 (77.2%) of Core Directors/Co-Directors provided assistance to investigators to generate preliminary data for new grant applications, 36 (63.2%) ensured that the technologies were up-to-date and addressed the needs of investigators, 30 (52.6%) professionalized staff positions and provided training opportunities, 27 (47.4%) implemented a cost-recovery system for equipment usage, 21 (36.8%) avoided unnecessary duplication of equipment, 19 (33.3%) have established a succession plan for Directorship, 13 (22.8%) have adopted a business plan for their core laboratories, and 3 (5.3%) adopted other strategies (Figure 7).

### 3.9. Annual Operating Costs of RCMI RIC Facilities

**Figure 8:**
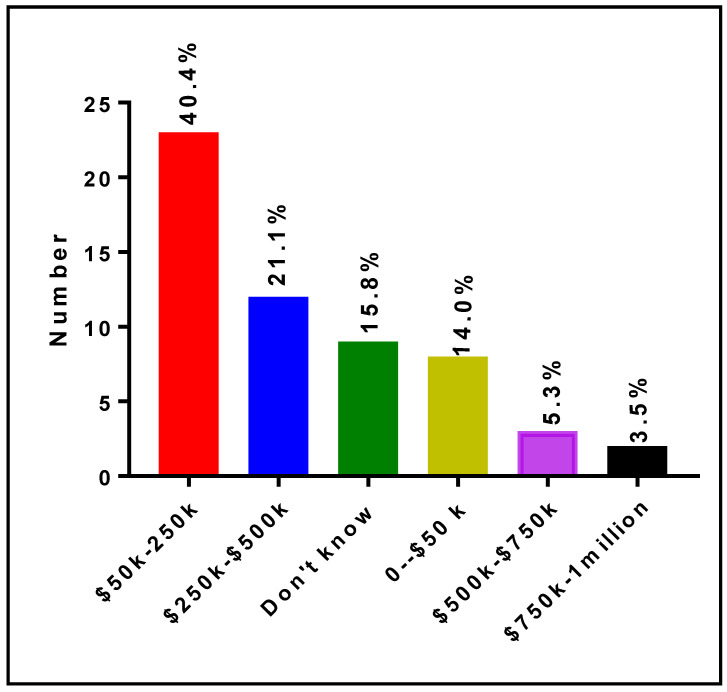
Annual operating costs of specific core laboratory at RCMI U54 Centers.

Figure 8 shows the responses of RIC Directors and Co-Directors on the yearly cost of operation of their core laboratories. It is indicated that 8 (14.0%), 23 (40.4%), 12 (21.1%), 3 (5.3%) and 2 (3.5%) Directors/Co-Directors estimate the operation costs to be between USD 0–USD 50,000, USD 50,000–USD 250,000, USD 250,000–USD 500,000, USD 500,000–USD 750,000, and USD 750,000–USD 1,000,000, respectively. In addition, nine (15.8%) stated that they did not know the estimated cost of operation of their core laboratories.

### 3.10. Funding Sources of RCMI RIC Facilities

**Figure 9:**
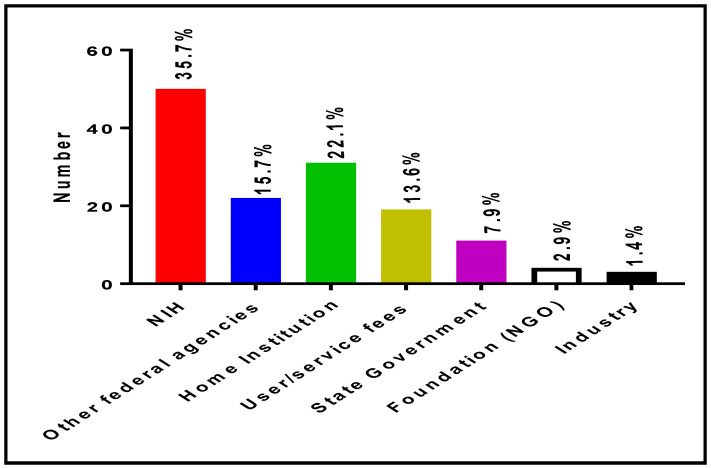
Funding sources of RIC facilities at RCMI U54 Centers.

Figure 9 shows the various sources of funding of RIC laboratories at RCMI U54 Centers. The data presented in this figure indicated that 35.7%, 15.7%, 22.1%, 13.6%, 7.9%, 2.9%, and 1.4% of funds needed for the operation of RIC laboratories come from the NIH, other federal agencies (National Science Foundation, Department of Defense, Department of Energy, etc.), home institutions, user service fees, state government, non governmental organizations, and industry, respectively.

### 3.11. Strategic Approaches Implemented by RIC Directors and Co-Directors to Manage the Core Research Facilities

**Figure 10:**
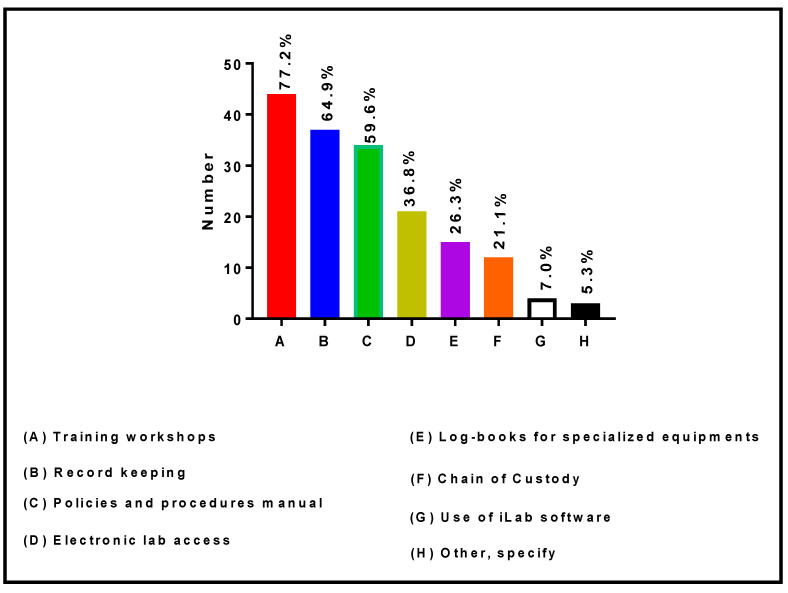
Strategic activities implemented by RIC Directors and Co-Directors to manage the core laboratories.

Figure 10 shows the strategic approaches implemented by RIC Directors and Co-Directors to manage the core research facilities. The data presented in this figure indicate that 77.2%, 64.9%, 59.6%, 36.8%, 26.3%, 21.1%, and 7.0% of RIC Directors and Co-Directors manage their core facilities through the organization of training workshops, record keeping, policies and procedures manuals, electronic lab access, logbooks for specialized equipment, chains of custody, and/or use of iLab software, respectively. In addition, three (5.3%) reported other strategic approaches, such as communication by word-of-mouth to other investigators via regular seminars and monthly administrative meetings.

### 3.12. Collaborations and Partnerships with Other NIH-Funded Programs/Networks to Leverage Research Resources and Maximize Productivity

**Figure 11:**
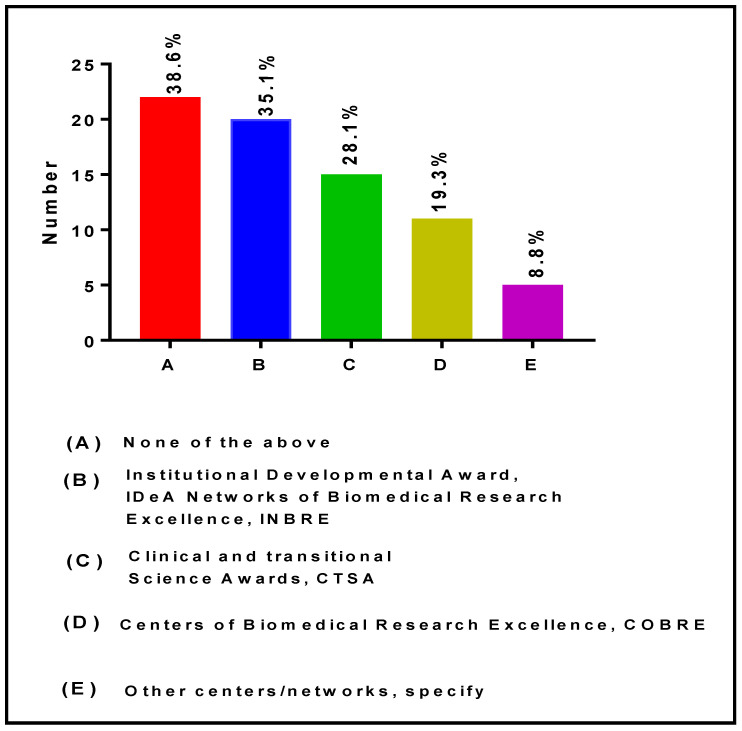
Collaborations and partnerships with other NIH-funded Centers and Networks.

Figure 11 shows the collaborations and partnerships with other NIH-funded Programs/Networks. The data presented in this figure indicate that 35.1%, 28.1%, 19.3%, and 8.8% of RIC Directors and Co-Directors have established collaborations and partnerships with CTSA, Institutional Developmental Award (IDeA) Networks of Biomedical Research Excellence (INBRE); Centers of Biomedical Research Excellence (COBRE), and/or other NIH Centers/Networks, respectively. Moreover, 22 (38.6%) reported that they do not have any collaboration with other NIH-funded Networks.

## 4. Discussion

RIC facilities are essential for the successful development and implementation of biomedical, socio-behavioral, clinical and/or translational research aimed at improving minority health and reducing or eliminating health disparities. These facilities provide access to specialized equipment and research resources needed by investigators to conduct high- quality state-of-the-art research [19]. Jeffries and colleagues [20] recently discussed the methodological approaches to understanding the causes of health disparities, and made a set of key recommendations toward research improvement, including: (1) Strengthen and promote analytic methods that maximize the ability to draw causal inferences from observational studies and enable a better understanding of health disparity causes; (2) Incorporate and further develop models that reflect the multi-level nature of health disparity causes to provide richer and more accurate characterizations of plausible causal pathways; (3) Expand the use of complex systems and simulation modeling to increase the ability to model intricate relationships between health disparities and health determinants, and to assess health disparities interventions; and (4) Incorporate the further use of qualitative and mixed methods analysis so participant perspectives can illuminate plausible causal mechanisms and provide a better understanding of the impacts of policies and interventions [18]. Meder and her research collaborators pointed out that the scientific advances leading to major breakthroughs in life sciences, such as the deciphering of human genome, stem cell therapy, and/or precision medicine, are inextricably linked to the technological advances and innovation in research infrastructure [19]. New scientific discoveries to improve human health will not be possible without the support of robust biomedical infrastructure. Our research indicates that the RIC facilities at the 21 RCMI U54 Centers are very well equipped to help the leaders of both research and pilot projects to implement their biomedical, socio-behavioral, clinical, and/or translational research on diseases that disproportionately affect minorities and underrepresented populations. By providing access to state-of-the-art equipment, scientific expertise, consultation services, and technology transfer, the RIC facilities play a key role in many important areas of biomedical, socio-behavioral, clinical and translational research [21,22].

From previous meetings conducted with the RIC Directors at RCMI U54 Centers, several impediments to the efficient operation and management of the core facilities were identified. Profound discussions from these meetings led to the full understanding of challenges and the development of potential solutions based on lessons learned. These barriers/obstacles and potential solutions are summarized in Table 4.

**Table 4:** Barriers to RIC facilities operation/management and potential solutions.

Impediments	Potential Solutions
Lack of sufficient funds for equipment service and maintenance contracts and/or for upgrades	Discuss with the institutional administrative leadership to return a percentage of indirect cost recovered back to the Center Develop and implement a clear policy of cost recovery for the use of specialized equipment Encourage faculty to include the cost of equipment usage in their grant proposals Develop and implement a marketing plan based on the strengths and opportunities provided by specific RIC facilities
Inadequate staffing of RIC laboratories	Hire qualified laboratory personnel Organize professional development workshops Sponsor personnel to participate in specialized training
Insufficient time and effort commitment	Request adequate protected time in the grant proposals Negotiate with the institutional administrative leadership for additional release time
Dearth of NIH-funded faculty to provide technical training and expertise in some areas of interest	Utilize the RCMI-CC Profiles database to identify scientific expertise Utilize the RCMI-CC eagle-i database to identify research resources Sponsor training opportunities to improve technical skills
Unavailability of some core technologies or equipment	Establish strong collaborations with other RCMI Centers and/or other NIH-funded Centers/Networks to leverage research resources and scientific expertise
Management-related issues in RIC facilities	Develop and implement an online/electronic scheduling system Continually assess the research resources needs of RCMI investigators Conduct SWOT analyses and develop and implement strategic/business plan, including a continuity of operations plan in the event of disruption of services Form regional alliances to facilitate close collaborations

One of the significant challenges for core facilities operation and management is for their Directors and Co-Directors to be able to adapt and cost-efficiently manage the ever-changing research landscape. Continuously building capacity in research infrastructure that is broadly applicable to a wide range of biomedical, socio-behavioral, clinical and translational researchers, is essential in fostering innovation leading to scientific discoveries in diseases that disproportionately affect minorities and underrepresented populations. Hence, it has become critically important to develop robust strategic plans for RIC facilities based on the vision for the future operation and the mission of specific core laboratories to address the biomedical research needs of RCMI investigators. Such RIC vision and mission should clearly align with RCMI institutions’ specific priorities, policies and procedures to maximize support and ensure the sustainability of biomedical research [23]. Leveraging the existing research technologies mainly depends on the scientific expertise of Core Directors and the technical staff who manage the RIC facilities on a day-to-day basis. As shown in Figure 4, RIC Directors, Co-Directors and other RIC personnel provide very high-level expertise and technical services to the research community. Hence, it is critically important to keep Core Directors and Co-Directors in tenure-track positions and institutionalize key technical staff’s jobs to limit frequent turnover, maintain stability, and maximize research productivity. According to the American Association of University Professors, the purpose of academic tenure is “to safeguard academic freedom, which is necessary for all who teach and conduct research in higher education.” Hence, it provides job security because tenured faculty can only be terminated either for cause or under extreme academic circumstances [24].

## 5. Limitations

The present research assessed the perspectives of RIC Directors and Co-Directors at 21 RCMI U54 Centers in the United States. Each of these 21 institutions has a RIC Director and 3–5 Co-Directors depending on the type/number of multi-users’ core laboratories that they operate. Hence, out the total of 98 RIC leaders, 21 are directors and the rest 77 are Co-Directors. Moreover, 57 of these leaders responded the survey, including 13 who self-identified themselves as directors and 44 as Co-Directors. Given the nature of the survey questionnaire and based on the informed consent which stated that “no personally identifiable information is included in the survey questionnaire”, it was not possible to link the respondents to their respective institutions. In addition, to avoid multiple responses from the same participant, the REDCap survey was set-up with a control mechanism that prevented an individual from responding to the survey more than one time. Although this survey research offered a cheaper method and practical way to gather data on the perspectives of RIC Directors and Co-Directors on the research resources and the management and operation of RIC facilities, this approach may be subject to bias from respondents who may provide dishonest answers to specific questions, and to differences on how the RIC leaders may understand the questions. To minimize these risks, appropriate steps were taken to ensure that the informed consent fully explained the rationale, goals and objectives, and significance of the RIC survey, the risks, and benefits of participation, and more importantly, to help them make an informed decision about their research participation.

Another limitation of this survey research is that it does not include questions related to the assessment of diversity, equity, and inclusion (DEI) in the questionnaire. Such questions would have provided useful information on the numbers and percentages of women, persons with disabilities, and underrepresented minority groups—Blacks or African Americans, Hispanics or Latinos, and American Indians or Alaska Natives—among the RIC Director and Co-Directors at RCMI U54 Centers. Broadening the participation of minority populations/under-represented groups through engagement in research and education is essential for not only developing a robust workforce in biomedical, socio-behavioral, and/or clinical sciences, but also for sustaining the United States preeminence as a global leader in research and development. According to the National Academies of Sciences, Engineering, and Medicine, DEI in the workplace is an asset that not only expands the available talent pool, but also increases the range of perspectives and expertise available to solve grand challenges in science, engineering, technology, engineering and mathematics [25,26].

## 6. Conclusions

Findings from the present study indicate that the RIC facilities are essential for the successful development and implementation of biomedical, socio-behavioral, clinical and/or translational research aimed at improving minority health and reducing or eliminating health disparities. At the RCMI U54 Centers, these facilities are equipped with state-of-the-art research resources to support the development and cost-effective implementation of scientific projects, including the research and pilot projects conducted by RCMI investigators. In addition to creating a robust research ecosystem that provides access to novel technologies, they also offer outstanding opportunities for multidisciplinary and transdisciplinary collaboration in biomedical, socio-behavioral, clinical and/or translational research. Although their operation and management suffer from several impediments primarily associated with financial constraints, Core Directors and Co-Directors are currently implementing a good number of strategic activities to improve efficiency and maximize research productivity.

## Figures and Tables

**Table 1:** Characteristics of specific R-series, P-series, and U-series funding mechanisms.

Funding Mechanisms	Characteristics
Research Project Grant Program (R01)	Used to support a discrete, specified, circumscribed research project NIH’s most commonly used grant program No specific dollar limit unless specified in FOA Advance permission required for USD 500 K or more (direct costs) in any year Generally awarded for 3–5 years
Small Grant Program (R03):	Provides limited funding for a short period of time to support a variety of types of projects, including pilot or feasibility studies, collection of preliminary data, secondary analysis of existing data, small, self-contained research projects, development of new research technology, etc. Limited to two years of funding Direct costs generally up to USD 50,000 per year Not renewable
NIH Academic Research Enhancement Award (AREA) (R15)	Supports small research projects in the biomedical and behavioral sciences conducted by undergraduate and/or graduate students and faculty in institutions of higher education that have not been major recipients of NIH research grant funds Limited eligibility Direct cost limited to USD 300,000 over entire project period Project period limited to up to 3 years
Exploratory/Developmental Research Grant Award (R21)	Encourages new, exploratory, and developmental research projects by providing support for the early stages of project development. Sometimes used for pilot and feasibility studies Limited to up to two years of funding Combined budget for direct costs for the two-year project period usually may not exceed USD 275,000 No preliminary data is generally required
Clinical Trial Planning Grant Program (R34)	Designed to permit early peer review of the rationale for the proposed clinical trial and support development of essential elements of a clinical trial Usually project period of one year, sometimes up to 3 years Usually, allows for a budget of up to USD 100,000 direct costs, sometimes up to USD 450,000
Research Program Project Grant (P01)	Support for integrated, multi-project research projects involving a number of independent investigators who share knowledge and common resources Each project contributes or is directly related to the common theme of the total research effort, thus forming a system of research activities and projects directed toward a well-defined research program goal Specific dollar limit unless specified in Funding Opportunity Announcement
Exploratory Grants (P20)	Often used to support planning activities associated with large multi-project program project grants
Center Core Grants (P30)	To support shared resources and facilities for categorical research by a number of investigators from different disciplines, who provide a multidisciplinary approach to a joint research effort or from the same discipline, and who focus on a common research problem The core grant is integrated with the left’s component projects or program projects, though funded independently from them
Specialized Centers (P50)	To support any part of the full range of research and development from very basic to clinical May involve ancillary supportive activities such as protracted patient care necessary to the primary research or R&D effort The spectrum of activities comprises a multidisciplinary attack on a specific disease entity or biomedical problem area Receives continuous attention from staff funding IC Centers may serve as regional or national resources for special research purposes
Research Project—Cooperative Agreements (U01)	To support a discrete, specified, circumscribed project to be performed by the named investigator(s) in an area representing his or her specific interest and competencies
Cooperative Clinical Research—Cooperative Agreements (U10)	To support clinical evaluation of various methods of therapy and/or prevention in specific disease areas These represent cooperative programs between sponsoring institutions and participating principal investigators, and are usually conducted under established protocols
Research Demonstration-Cooperative Agreements (U18)	To provide support for testing, by means of a research design, the effectiveness of the transfer and application of techniques or interventions derived from a research base for the control of diseases or disorders or for the promotion of health The project should be capable of making conclusions which are generalizable to other sites These are usually cooperative programs between participating principal investigators, institutions, and the sponsoring Institute(s)
Research Program—Cooperative Agreements (U19)	To support a research program of multiple projects directed toward a specific major objective, basic theme or program goal, requiring a broadly based, multidisciplinary, and often long-term approach A cooperative agreement research program generally involves the organized efforts of large groups, members of which are conducting research projects designed to elucidate the various aspects of a specific objective Substantial federal programmatic staff involvement is intended to assist investigators during performance of the research activities, as defined in the terms and conditions of award. The investigators have primary authorities and responsibilities to define research objectives and approaches, and to plan, conduct, analyze, and publish results, interpretations and conclusions of their studies Each research project is usually under the leadership of an established investigator in an area representing his/her special interest and competencies Each project supported through this mechanism should contribute to or be directly related to the common theme of the total research effort The award can provide support for certain basic shared resources, including clinical components, which facilitate the total research effort These scientifically meritorious projects should demonstrate an essential element of unity and interdependence
Special Cooperative Investigations/Assessment of Control/Prevention Methods (U50)	In cooperation with State or local government or other health-related organizations, to perform special investigations of communicable diseases and other preventable health conditions, or to evaluate special methods of preventing disease or controlling health conditions
Specialized Center—Cooperative Agreements (U54)	To support any part of the full range of research and development from very basic to clinical; may involve ancillary supportive activities such as protracted patient care necessary to the primary research or R&D effort The spectrum of activities comprises a multidisciplinary attack on a specific disease entity or biomedical problem area These differ from program project in that they are usually developed in response to an announcement of the programmatic needs of an Institute or Division and subsequently receive continuous attention from its staff Centers may also serve as regional or national resources for special research purposes, with funding component staff helping to identify appropriate priority needs
Cooperative Agreements in Occupational Safety and Health Research, Demonstrations, Evaluation and Education Research, Demonstrations, Evaluation and Education (U60)	In cooperation with universities or other eligible recipients, to investigate the underlying characteristics and causes of occupational safety and health problems To eliminate or control factors in the work environment which are harmful to the health and/or safety of workers, or To demonstrate effective solutions for occupational safety and health problems

**Table 2:** Major research resources available in the Research Infrastructure Core facilities at the 21 RCMI U54 Centers.

**Analytical Chemistry**
Costech model 4010 Elemental Combustion System (Elemental Analyzer) Malvern Instruments Model Zetasizer model 3600 (Zen3600/MPT-2 system) Shimadzu Model AA-6701 atomic absorption spectrometer Shimadzu model UV-2600 UV-Vis spectrophotometer with temperature control accessory Shimadzu Model UV-3700 Solid Spec UV-VIS-NIR spectrophotometer Nicolet Model Nexus 870 FT-IR Spectrometer/Auto Image System Shimadzu Model 17A GC/ECD (Gas Chromatograph/Electron Capture Detector) Spex Model Raman Spectrometer with microscope Instruments S.A. Model FluoroMax-2 Spectrofluorometer Horiba model FluoroMax4 fluorescence spectrometer with 200 ps TCSP (time-resolved) Amersham Biosciences ÄKTA FPLC (Model 18-1118-67) with Fraction Collector HP (Hewlett Packard) Model 6890/5973 GC-MS (Gas Chromatography/Mass Spectrometer Agilent 7100 Capillary Electrophoresis system Shimadzu prominence 2020 HPLC with UV and Fluorescence detectors Thermo Finnigan TSQ Quantum HPLC-MS system Finnigan LCQ DECA mass spectrometer with NANO Spray Ion Source Varian Model 820-MS ICP-MS system Agilent 7800 ICP-MS Agilent 6546 Q-TOF LC/MS System
**Animal Care and Animal Research**
Large-scale cage washers Large-size −20 °C freezers Humidity and temperature control equipment Various regular racks and cages for rats and mice Special metabolic cages A ventilation system Food and water dispensers Food and bedding storage and a general storage area
**Behavioral Research**
Computer workstations Data analysis software packages, such as STATA 15.0 and SPSS 24, SAS 9.4, atlas.ti and MAXQDA Geographic Information System (ArcGIS) Research design and methodology; Information analysis: statistical (quantitative), qualitative methodological design and analysis, informatics; Data Management (storage and security); and Data Access infrastructure REDCap surveys and evaluation of projects outcomes
**Bioinformatics and Computational Biology/Modeling**
Dell Precision Tower 7910 workstations with up to 64 GB RAM Linux servers (Ubuntu 18.04LTS) containing 128 GB RAM and GPUs with more than 500 streaming processors for supercomputing and bioinformatics research Multiple server nodes Supercomputing power Management of bioinformatics databases, molecular modeling and simulations, next generation sequencing (NGS) and microarray datasets, genome-wide SNP and eQTL analyses, and biomarkers discovery for disease diagnosis and prognosis Advanced Cyberinfrastructure Coordination Ecosystem: Services and Support (ACCESS; https://access-ci.org/ accessed on 10 October 2022).
**Biostatistics and Study Design**
Dell OptiPlex desktop computers and several laptops with Intel core i7 processors running on Windows 10 operating system All computers carry latest versions of statistical and modeling software, such as SAS (with several modules such as SAS/STAT; SAS/ASSISt, etc.), SPSS, SysStat, Minitab, Neuroshell, @Risk, and Matlab NGS analysis software for gene network and pathway analysis Statistical analysis and data normalization will be carried out with software package JTouch 65-inch whiteboard with capacitive touch and overhead projectors for training and presentations
**Clinical Research Resources**
Clinical Research Nursing Services (protocol management including case report form [CRF] development, patient screening, scheduling and management of study visits) REDCap surveys; Phlebotomy; Specimen Processing, Storage and Shipment Body Composition and Fitness and other anthropomorphic measurements, bone density and whole body (fat, lean tissue) composition measurements, Neuropsychological Testing Point of Care Services (portable assessment of lipid [total, HDL cholesterol, triglycerides], hemoglobin A1c [HbA1c], CRP) Fourier Transform-Infrared Spectroscopy [FTIR] (assessment of total body water) Research clinic rooms Small equipment (blood pressure equipment, temperature, etc.)
**Confocal Microscopy**
Zeiss LSM 700 confocal and imaging analysis system Olympus FV1000 confocal laser scanning microscope system with an inverted IX81 microscope Leica SP2 Confocal Microscopy System Leica STELLARIS 5 Confocal Microscope Leica SP5 Confocal Microscope
**Data Science and Informatics**
High performing computer servers Licensed and open-source software and frameworks QIAGEN CLC Genomics Workbench 20^©^ QIAGEN Ingenuity Pathway Analysis^©^ QIAGEN Ingenuity Variant Analysis^©^ R and R-Studio software Python, Anaconda, Jupyter, Panda, Scit-Learn, R, phpMyAdmin, JavaScript, PHP, Drupal, DSpace, Plone, and other programs REDCap
**Electron Microscopy**
JEOL-1011 Transmission Electron Microscope system equipped with a 0.2 nm lattice resolution with magnification of 50 to 1,000,000 under the accelerating voltage of 40 to 100 kV, a camera system, and a computer with LC monitor JEOL JEM-1400 transmission electron microscope with 120 kV accelerating voltage; electron gun assembly with Cool Beam Illumination System–LaB6 filament standard with a comprehensive Energy Dispersive X-ray Microanalysis system Quanta 200 Environmental Scanning Electron Microscope (ESEM) equipped with a CCD IR camera and a Pentium PC with LCD monitor Tuscan LYRA3 Focused Ion Beam Scanning Electron Microscopy system (SEM/FIB) equipped with 2 closed loop nano-manipulators and an electron dispersive X-Ray spectroscopy analyzer (EDX) for elemental analysis
**Flow Cytometry**
Becton Dickinson Biosciences Fluorescence Activated Cell Sorting System-FACS Calibur Becton Dickinson Biosciences Fluorescence Activated Cell Sorting System-FACS Lyric Beckton-Dickinson FACSAria II Flow Cytometer/Sorter, with BioBubble BSL2 aerosol enclosure Beckman-Coulter Moflo Astrios Fluorescence Activated Cell Sorting system Becton Dickinson FACSVerse Beckman-Coulter Moflo Gallios Flow Cytometer ThermoFisher Attune NxT Flow Cytometer with autosampler SONY SH800 cell sorter SONY SA3800 Spectral analyzer
**Genomics and Gene Sequencing**
Illumina RNASeq 550 SystemIllumina NextSeq 2000 Illumina MiSeq Applied Biosystems 3500 Genetic Analyzer Applied Biosystems 7500 Fast Dx Real-Time PCR system Applied Biosystems StepOne Plus Real-Time PCR system Agilent Technologies 4200 TapeStation electrophoresis system Automatic environmental speed Vac system BioRad molecular image gel documentation system Eppendorf master cycler gradient thermocycler, PCR Workstation, Stratagene Stratalinker 1800 UV crosslinker BioRad gel dryer, BioRad UV spectrophotometer, Precision incubator/oven, Omni tissue homogenizer, electrophoresis units, Amersham electro-blotting system Olympus inverted phase contrast microscope with camera, Affymetrix Microarray system/array scanner (Affymetrix G250) Olympus epifluorescence microscope with camera BioRad microplate reader Labconco™ FreeZone™ Freeze Dry System Beckman ultracentrifuge
**Histology and Pathology**
Leica Laser Dissection Microscopes Leica Aperio CS2 DAKO Autostainer Link 48 Zeiss LSM 700 Confocal Microscope Leica Aperio VERSA Vacuum Impregnation System Paraffin Embedding Center with wax drum, cooler and heating chamber High-Capacity Section Dryer and Slide Drying Hotplate with automatic temperature control Leica CM 1900 and 1950 Advanced Clinical Cryostat Leica TP 1020 Automatic Tissue Processors Leica RM 2135 Rotary Microtomes and Leica SM 2003 and 2010R Sliding microtomes Leica EG 1150 Tissue-Embedding Centers Primera Slide Printers
**Mass Spectrometry**
Thermo Model TSQ Quantum GC-MS/MS /LC-MS/MS Varian Model 820-MS LC-ICP-MS Maldi-TOF Mass Spec, Q-Exactive Plus, UltiMate 3000 RS Autosampler, UltiMate 3000 RSLCnano System, UltiMate 300 Solvent Holder, UltiMate 3000 RS pump, UltiMate 3000 Solvent Holder, Linear Ion trap LTQ XL, Nanomate NMT-TV Triversa, nanoLC-1D Plus, autosampler to nanoLC-1D Plus Trace 1310—GCMS Mass Spec, TSQ 9000, GSMS autosampler—AI1310, TSQ Endura, UltiMate 3000 RS Column Compartment, UltiMate 3000 RS Autosampler, UltiMate 3000 RS Pump, UltiMate 3000 Solvent Holder, UltiMate 3000 Automated Fraction Collector, Corona Veo RS, Furnace (1200 Celsius) for glycomics Sciex 6500 Triple Quad and 7500 Triple Quad LC-MS/MS systems Sciex 500B QTOF system PELCO Pella BioWavePro Microwave System Leica UC7 ultramicrotome Leica CM1860 cryostat
**Microscopy and Biomedical Imaging**
Bruker MRI equipment systems LC Model for analysis of in vivo MRS data Bruker Albira Si, capable of small animal positron emission tomography (PET), single photon emission computed tomography (SPECT) and computed tomography (CT)Bruker acquisition/reconstruction workstation with image processing PMOD software ImageXpress Pico Automated Cell Imaging System (Molecular Devices). BD Pathway 855 BioImager Molecular Devices. ImageXpress Pico Automated Cell Imaging System Two Zeiss LSM 700 confocal microscopes Zeiss FRET software module for ZEN 2009 Leica CM1900 Cryostat Aperio CS2 Scanner system Optical imaging machine (PerkinElmer IVIS Spectrum) with high-sensitivity, high throughput of fluorescence and bioluminescence in vivo imaging Olympus FV1000 confocal laser scanning microscope system with an inverted IX81 microscope featuring improved basic performances Leica TCS SP2 Confocal microscopy system that uses high intensity laser light and a confocal aperture to obtain optically focused images from fluorescent samples Olympus Epifluorescence microscopy system equipped with Comet assay software for DNA damage analysis Nikon 90i and TE2000-S fluorescence microscopes Keyence BZ-XB fluorescence microscope
**Molecular Biology**
Automatic Environmental Speed Vac System (Savant) Labconco™ FreeZone™ Console Freeze Dry System (Labconco) Cryosafe Liquid Nitrogen Tank (Forma Scientific) Molecular Image Gel Documentation System (BioRad) Nucleic Acid Sequencing System and Accessories (Bio Rad) Mastercycler Gradient Thermocycler (Eppendorf Scientific) Konico SRX-101 automatic film processor and densitometer (Mid-South) Biosafety Cabinet with UV (Forma Scientific) PCR Workstation (Air Clean Systems) Stratalinker 1800 UV Crosslinker (Stratagene) Power Plus Upright Freezers (Forma Scientific) UV Spectrophotometer (BioRad) Refrigerator (4 °C glass door) (Fisher) Microwave (Goldstar) Incubator/oven (Napco) Scotsman ice maker (Fisher) Tissue Homogenizer (Omni) Horizontal Electrophoresis Apparatus (BioRad) Vertical Electrophoresis Apparatus (Amersham) Electro-Blotting Transfer System (Amersham) Olympus Inverted Phase Contrast Microscope with Camera (C-Squared) Analytical Balance (Fisher) Refrigerated Microcentrifuge 4 °C (Forma Scientific) pH Meters (Fisher) Precision 37 °C Incubator (Fisher) Humidified CO_2_ Incubators (Sanyo) Sonifer Cell Disruptor Model 250/450 (VWR Scientific) Olympus Epifluorescence Microscope Nikon E-600 Imaging System with Camera ©-Squared) Water Purification System (Barnstead) CEQ 8000 Genetic Analysis System (Beckman Coulter) StepOne RT-PCR System (Life Technologies—Thermo Fisher) Illumina RNQSeq 550 (NextSeq 550) System Phosphoimagers
**Nuclear Magnetic Resonance Spectroscopy**
Bruker 500 MHz NMR spectroscopy system with analytical probes NMR spectrometer-Varian 500 MHz NMR spectrometer-Bruker DMX 300 MHz EPR Spectrometer-Bruker EMX
**Proteomics**
Shimadzu Model AA-6701 atomic absorption spectrometer Nicolet Model Nexus 870 FT-IR Spectrometer/Auto Image System Shimadzu Model 17A GC/ECD (Gas Chromatograph/Electron Capture Detector) Spex Model Raman Spectrometer with microscope Horiba model FluoroMax4 fluorescence spectrometer with 200 ps TCSP (time-resolved) Hewlett Packard Model 6890/5973 GC-MS (Gas Chromatography/Mass Spectrometer Shimadzu prominence 2020 HPLC with UV and Fluorescence detectors Thermo Finnigan TSQ Quantum HPLC-MS system Finnigan LCQ DECA mass spectrometer with NANO Spray Ion Source Varian Model 820-MS Thermo Easy n-LC 1200 coupled to the Thermo Q-Exactive Plus Mass Spectrometer Varioskan Flash Spectral Reader (Thermo Scientific) Chemi Doc XSR+ (Bio Rad) Turbo Trans Blot Transfer System (Bio Rad) Sorvall WX Ultra Series Ultracentrifuge (Thermo Scientific) miVac DNA Concentrator Integrated system and Savant Speed Vac Plus Thermo LTQ Orbitrap XL Thin Layer electrophoresis NANODROP 2000 (Thermo Scientific) Glomax Multi Detection System (Promega) iMark Microplate Reader (BIO-RAD) Proteome Discovery 1.4 and 2.4 Software SIEVE 2.1 Ingenuity Pathway Analysis
**Tissue and Cell Culture**
In-situ −4 °C cold rooms Labconco™ FreeZone™ Console Freeze Dry System (Labconco) Cryosafe Liquid Nitrogen Tank (Forma Scientific) Power Plus Upright Freezers (Forma Scientific) UV Spectrophotometer (BioRad) Refrigerators/Freezers Combos (Fisher) Incubator/oven (Napco) Tissue Homogenizer (Omni) Olympus Inverted Phase Contrast Microscope with Camera (C-Squared) Analytical Balances (Fisher) Refrigerated Microcentrifuge 4 °C (Forma Scientific) Precision 37 °C Incubators (Fisher) Humidified CO_2_ Incubators (Sanyo) Olympus Epifluorescence Microscope Nikon E-600 Imaging System with Camera ©-Squared) Water Purification System (Barnstead) Laminar flow Biosafety Cabinets Cryogenic Storage Tanks Heater Water Baths

## Data Availability

All data generated from this research are presented in this manuscript.
